# Berberine Suppresses Colonic Inflammation in Dextran Sulfate Sodium–Induced Murine Colitis Through Inhibition of Cytosolic Phospholipase A2 Activity

**DOI:** 10.3389/fphar.2020.576496

**Published:** 2020-11-19

**Authors:** Lixiang Zhai, Tao Huang, Hai-tao Xiao, Pei-gen Wu, Cheng-yuan Lin, Zi-wan Ning, Ling Zhao, Hiu Yee Anna Kwan, Xian-jing Hu, Hoi Leong Xavier Wong, Xian-qian Li, Zhao-xiang Bian

**Affiliations:** ^1^ School of Chinese Medicine, Hong Kong Baptist University, Kowloon, Hong Kong; ^2^ School of Pharmacy, Health Science Center, Shenzhen University, Shenzhen, China; ^3^ School of Pharmaceutical Sciences, Guizhou Medical University, Guiyang, China; ^4^ Shenzhen Research Institute and Continuing Education, Hong Kong Baptist University, Shenzhen, China

**Keywords:** ulcerative colitis, macrophage, phospholipase A2, lysophosphatidylcholine, berberine

## Abstract

Ulcerative colitis (UC) causes chronic inflammation and damage to the colonic mucosal layer. Recent studies have reported significant changes in phosphatidylcholine (PC) and lysophosphatidylcholine (LPC) in UC patients and oral administration of PC has considerable therapeutic effects against UC, suggesting the metabolism of phosphatidylcholine may be involved in the UC development. Our previous work has demonstrated that berberine effectively suppresses inflammation and protects colonic mucosa injury in DSS-induced colitic mice. However, whether the therapeutic effects of berberine are attributed to its action on the PC metabolism remains unknown. In the present study, we have shown that berberine significantly reduces the lysophosphatidylcholine (LPC) levels in the sera of DSS-induced experimental colitis mice and LPS-stimulated macrophage RAW 264.7 cells. The cytosolic phospholipase A2a (PLA2G4A), an enzyme for hydrolyzing PC to LPC, was found to be up-regulated in the colon tissue of experimental colitis mice and inflamed macrophage RAW 264.7 cells. We then demonstrated berberine inhibits the phosphorylation of cytosolic phospholipase A2a (PLA2G4A) in the colon tissue of experimental colitis mice and inflamed macrophage RAW 264.7 cells. Subsequently, we revealed berberine suppressed the expression of pro-inflammatory factors including TNF-alpha and IL-6 through regulating PLA2G4A dysfunction in macrophage RAW 264.7 cells. Mechanistically, we found that berberine directly binds to PLA2G4A and inhibits MAPK/JNK signaling pathway to inhibit PLA2G4A activity in inflammatory status. Therefore, we concluded that berberine inhibits colonic PLA2G4A activity to ameliorate colonic inflammation in experimental colitic mice, suggesting modulation of the PC metabolism via PLA2G4A might be beneficial for establishing new therapies strategy for UC.

## Introduction

Ulcerative colitis (UC) and Crohn’s disease (CD) are classified as severe and chronic inflammatory bowel disease (IBD) that causes inflammation and sores (ulcers) in the gastrointestinal tract, particularly in the large intestine (Kaplan, 2015). Lipid metabolism is in relation to chronic inflammation as one of the possible mechanisms involved in the pathogenesis of many inflammatory diseases (Parhofer, 2015). Notably, IBD patients have been found with dyslipidemia by several studies, suggesting lipid metabolism and signaling may play key roles in modulating inflammation in IBD (Biyyani et al., 2010; Karaahmet et al., 2013). In lipid metabolism and signaling, phosphatidylcholine (PC) is a phospholipid that has been shown to account for more than 70% of total phospholipids in the intestinal mucus layer. UC patients were found to have decreased PC content, while the clinical trial studies revealed PC supplementation to the colonic mucus alleviated the inflammation in UC patients. In contrast, lysophosphatidylcholine (LPC) is derived from PC which is hydrolyzed by phospholipase A2 in the circulation. LPC has been reported with pro-inflammatory (Olofsson et al., 2008; Qin et al., 2014) properties in macrophages and apoptotic effects on vascular endothelial cells (Aiyar et al., 2007; Song et al., 2010). Moreover, lipidomics investigation on IBD patients has revealed alterations of lysophosphatidylcholine (LPC) (Chen et al., 2008; Chander et al., 2014; Fan et al., 2015). Therefore, the PC and LPC alterations in lipid metabolism and signaling may be involved in the colonic inflammatory responses in IBD, and therapeutic approaches regulating the PC and LPC metabolism or its related enzymes are potentially beneficial for suppressing inflammation in colitis.

Berberine is a natural benzylisoquinoline alkaloid found in a wide variety of plants such as *Coptis chinensis* Franch. (Chinese goldthread), *Berberis vulgaris* L. (European barberry), and *Berberis aquifolium* Pursh. (Oregon grape). Recent studies along with our findings have suggested berberine alleviates experimental colitis by suppressing inflammatory responses in macrophages and T cells and maintaining intestinal barrier function (Li et al., 2015; Li et al., 2016; Zhang et al., 2017; Liu et al., 2018) via regulation of multiple signaling pathways (Cui et al., 2009; Jeong et al., 2009; Amasheh et al., 2010; Zhou et al., 2015). Besides, berberine exhibits modulatory abilities on phospholipid metabolism in metabolic diseases such as obesity and non-fatty liver disease (Zhang et al., 2011; Hu et al., 2012; Wei et al., 2016; Zhao et al., 2017). Since phospholipid metabolism plays an important role in IBD, we wondered whether the pharmacological mechanism of berberine action on IBD involves phospholipid metabolism and signaling. For this purpose, we first characterized the berberine action on phospholipid metabolism in an experimental colitis mice model. Second, we determined whether the anti-inflammatory effects of berberine in animal models of colitis are mediated by modulation of phospholipid-related targets.

## Materials and Methods

### Regents

Berberine chloride was purchased from Shenzhen ChemStrong Scientific Co., Ltd. (Shenzhen, China) at the highest available purity (≥95%). Lipopolysaccharide (LPS), TNF-α, and endotoxin removal solution were purchased from SIGMA (St. Louis, MO, United States). Interferon-γ was obtained from Merck (EMD Millipore, MA, United States). cPLAα, p-cPLAα, JNK, and p-JNK antibodies were purchased from Cell Signaling (Danvers, MA, United States). Insulin was purchased from Life Technologies (Grand Island, NY, United States). Lipid standards, including SPLASH^™^ Lipidomix^®^ Mass Spec Standard, and LPC standards were purchased from Avanti Polar Lipids (Alabaster, AL, United States). PLA2 activator, JNK inhibitor and activator, Mouse *Pla2g4*, and control siRNA were purchased from Santacruz (Dallas, TX, United States). The recombinant human PLA2G4A protein was obtained from R&D Systems (Minneapolis, MN, United States).

### Cell Culture

RAW 264.7 macrophage cell line was obtained from ATCC (Manassas, VA, United States). RAW 264.7 cells were grown in DMEM (Gibco) supplemented with 10% fetal bovine serum (Gibco). RAW 264.7 cells were maintained at 37°C in a humidified atmosphere containing 5% CO_2_. Berberine hydrochloride was dissolved in DMSO to prepare 50 mM stock solution. Other reagents were dissolved in 1× phosphate-buffered saline (PBS) as stock solution.

### Animal Experiment

C57BL/6 background male mice were purchased from the Chinese University of Hong Kong. They were maintained in the animal laboratory of the School of Chinese Medicine, Hong Kong Baptist University (HKBU). The animals were kept in a standard living condition (room temperature 22°C, 12-h light/dark normal cycle, and constant humidity) with diet and water *ad libitum*. All experimental protocols and procedures were supervised by the Animal Care Ethics Committee of HKBU and the Department of Health, Hong Kong Special Administrative Region. Induction of colitis on mice was through 2.0% (w/v) dextran sulfate sodium (DSS) in drinking water. After 3 days of development, colitis mice were confirmed by fecal occult blood testing and randomly divided into two groups (model group and berberine treatment group). Mice in the berberine treatment group were administered berberine hydrochloride solution (suspended in water, dosage = 20 mg/kg) through oral gavage, while mice in the model group were administered water as parallel control. On day 7, the mice were anesthetized by 3% chloral hydrate through intraperitoneal injection. About 1 ml serum was collected through hemostasis from the heart to sacrifice the mice.

## Lipidomics Study

### Lipidomics Profiling on Mice Serum and Colon Tissue

For lipidomics study on serum, 30 μL serum and 30 μL water were added with 240 μL Folch solvent (chloroform:methanol = 2:1) and vortexed vigorously. Two phases were formed after centrifugation at 8,000 rpm for 15 min at 4°C. For lipidomics study on colon tissue, about 10 mg frozen colon tissue was weighed and homogenized in 1× PBS with a 1:10 m/v ratio. Then, 60 μL homogenate was added with 240 μL Folch solvent (with internal standard) for lipid extraction. The samples were vortexed and centrifuged at 8,000 rpm for 15 min at 4°C to induce phase separation. About 150 μL down layer solution was collected and dried under a vacuum concentrator within 30 min at room temperature. The samples were stored in −80°C until analysis.

### Lipidomics Profiling on RAW Cell Lines

RAW 264.7 cells were extracted according to a well-agreed protocol from LIPID MAPS using Bligh–Dyer (BD) extraction. In brief, cells from a 100-mm culture plate were washed twice with 5 ml ice-cold 1× PBS after aspirating the culture medium. Then, 10 ml 1× PBS was added to the plate, and the cells were scraped and transferred to 15-ml conical tubes. Then, 200 μL suspension was taken out for protein quantification by a protein BCA kit. Cell pellets were collected after centrifugation at 3,500 rpm for 10 min at 4°C. Then, the BD system: 1 ml of 80°C pre-cold 70% MeOH, 350 μL CHCl_3_, 350 μL H_2_O, and 350 μL CHCl_3_ were added step by step and vortexed at each step to induce phase separation. After centrifugation at 4,000 rpm for 30 min at 4°C, the down layer (200 μL) was transferred to 1.5 ml tubes. The samples were dried under vacuum at room temperature within 30 min and stored at −80°C until analysis.

Before LC-MS analysis, the dried samples were dissolved with 200 μL of acetonitrile/isopropanol/H_2_O (65:30:5, v/v/v) and centrifuged at 14,000 rpm for 10 min at 4°C. For LC-MS analysis, the liquid chromatogram was performed on an Agilent 1290 UPLC system and mass spectrometry was employed on an Agilent 6540 quadrupole time-of-flight mass spectrometry system. The Waters ACQUITY UPLC HSS C18 column (2.1 mm × 100 mm, 1.8-μm particle size) was used for separation at 40°C. Mobile phases A and B were prepared as 60:40 water/acetonitrile and 90:10 isopropanol/acetonitrile with 10 mM ammonium formate and 0.1% formic acid. The linear gradient was set as 0–32% B (0–0.8 min), 32–45% B (0.8–2 min), 45–52% B (2–2.5 min), 52–58% B (2.5–4 min), 58–66% B (5–5.5 min), 66–70% (5.5–7 min), 70–75% B (7–8.8 min), and 75–97% B (8.8–10.5 min); then maintained at 97% B from 10.5 to 15 min; and decreased to 32% B from 15 to 18 min. Lipids were detected and identified in both negative and positive ionization modes. The injection volume is 3 μL in the negative mode and 1 μL in the positive mode for each analysis. For lipid identification, the frame m/z values were used to search for information on LIPID MAPS and Human Metabolome Database (HMDB). The matches were confirmed by exact tandem mass (MS/MS) of lipids and retention time based on standards and databases.

### Transfection Study on RAW Cell Lines

Neon^®^ Transfection System (Life Technologies) was used following the instructions of the manufacturer for transfection study. For transfection on RAW 264.7 cells, 1 × 10^6^ cells were transfected with 20 nM siRNA with one pulse of 1,680 V for 20 ms. After transfection, cells were seeded in a 6-well cell culture plate to recover overnight in antibiotics-free medium. Then, RAW 264.7 cells were incubated with berberine 10 μM for 2 h before challenged with stimuli (LPS) for 24 h. Mouse *Pla2g4* siRNA and matched control siRNA were transfected using the Neon Transfection System according to the manufacturer’s instructions. Quantitative RT-PCR was used to detect transfection efficiency.

### Quantitative RT-PCR Analysis

Total RNA was isolated from RAW 264.7 cells using TRIzol reagent (TAKARA, Japan) following the manufacturers’ instruction, and cDNA was synthesized with a reverse transcriptase kit (TAKARA, Japan). For RT-PCR analysis, amplification was performed using SYBR Green for 40 cycles (94°C for 30 s, 57°C for 20 s, and 72°C for 20 s). The primers were designed and synthesized from Invitrogen. The primer sequences are provided in Supplementary Table S1 (Ashley et al., 2016).

### Western Blot Analysis

An equal amount of protein (10 μg) from each sample was separated on SDS-PAGE gels and transferred to nitrocellulose membranes. Blots were blocked with 5% non-fat milk in Tris-buffered saline with 0.1% Tween-20 (TBST, 25 mM Tris, pH 8.0, 137 mM NaCl, 2.7 mM KCl, and 0.1% Tween-20) at room temperature for 1 h, followed by overnight incubation with primary antibodies at 4°C. The primary antibodies were prepared with 5% bovine serum albumin (BSA). After being washed with TBST five times, the blots were hybridized with secondary antibodies conjugated with horseradish peroxidase (Bio-Rad, Hercules, CA, United States) in 5% non-fat milk dissolved in TBST at room temperature for 1 h and washed five times with TBST. The membranes were then incubated with enhanced chemiluminescence reagents and exposed to X-ray film (Fuji, Japan).

### Nitric Oxide and ELISA Assay

NO production was evaluated measuring nitrite, the stable metabolite of NO, from the culture media of RAW 264.7 by a modified Griess reagent (Sigma) according to the manufacturers' instructions. Duplicates of 100 μL of supernatant or culture media were added to 96-well microtiter plates and mixed with 100 μL Griess solution. The plate was then read on a microtiter plate reader using 540-nm wavelength. TNF-α and IL-10 were measured from supernatants of RAW 264.7 cells through enzyme-linked immunosorbent assay (ELISA) analysis by using ELISA kits according to the manufacturer’s protocol (eBioscience, San Diego, CA, United States). Duplicates of 100 μL of supernatant or culture media were added to 96-well coating plates for ELISA analysis using 570-nm wavelength. The result obtained from the microtiter plate reader was normalized by measured protein.

### Human PLA2G4A Assay

The PLA2G4A activity was measured by its ability to hydrolyze 1-hexadecanoyl-2-(1-pyrene-decanoyl)-sn-glycero-3-phosphocholine. The berberine was dissolved in DMSO (50 mM) as a stock solution to prepare the enzyme–inhibitor complex. The assay procedure was followed by the protocol online from R&D Systems.

### Docking Study of PLA2G4A and Berberine

Homology modeling of “open lid” conformation of PLA2G4A: The “open lid” conformation of PLA2G4A catalytic domain was constructed by grafting the “open lid” in PLA2G4D (PDB ID: 5IXC) onto the “closed lid” structure of PLA2G4A (PDB ID: 1CJY). First, the catalytic domain structures of PLA2G4A (residue 141–749) and PLA2G4D (residue 277–810) were superposed based on main-chain atoms and refined with Gaussian distance weights in Molecular Operating Environment (MOE) version 2012.10 (Deeth et al., 2005; Maier and Labute, 2014). Second, the lid region in PLA2G4D and residue 530–575 were selected and used as override template. Third, the homology model of the “open lid” conformation of PLA2G4A was built with 1CJY as the main template and residue 530–575 in 5IXC as an override template, besides of using methyl gamma-linolenyl fluorophosphonate as environment ligand in MOE. Finally, the model was optimized with AMBER10: EHT force field and scored by GB/VI function.

Docking of berberine into the “open lid” conformation of PLA2G4A: The 3D structure of berberine was extracted from the crystal structure of the QacR–berberine complex (PDB ID: 3BTI). The whole docking study was performed with MOE: dock. The methyl gamma-linolenyl fluorophosphonate was used as a reference ligand to guide the berberine docking into the active site of PLA2G4A. The placement method was Triangle Matcher, and a flexible receptor was used during the refinement stage. The GBVI/WAS dG was selected as the final scoring function. Docking poses were ranked by the GBVI/WAS dG score and analyzed by manual observation as well as ligand interactions modules. All 3D structures were drawn with VMD 1.8.3 software (Humphrey et al., 1996).

### Statistical Analysis

Results were from multiple, at least three times independent experiments. The lipidomics data were processed by the R package with XCMS. MetaboAnalyst was used for multivariate statistical analysis. Cytoscape was used to match the associated enzyme in the KEGG database. ImageJ was used to process Western blot results. Other data are expressed as average and SD value of at least triplicate samples. Significance *p* values were calculated using GraphPad Prism 6 from either Students’ *t*-test or one-way ANOVA, and the *p* value less than 0.05 was regarded as statistically significant.

## Results

### Berberine Reduces Lysophosphatidylcholine Level in Dextran Sulfate Sodium–Treated Colitis and Lipopolysaccharides-Stimulated RAW 264.7 Cells

**FIGURE 1:**
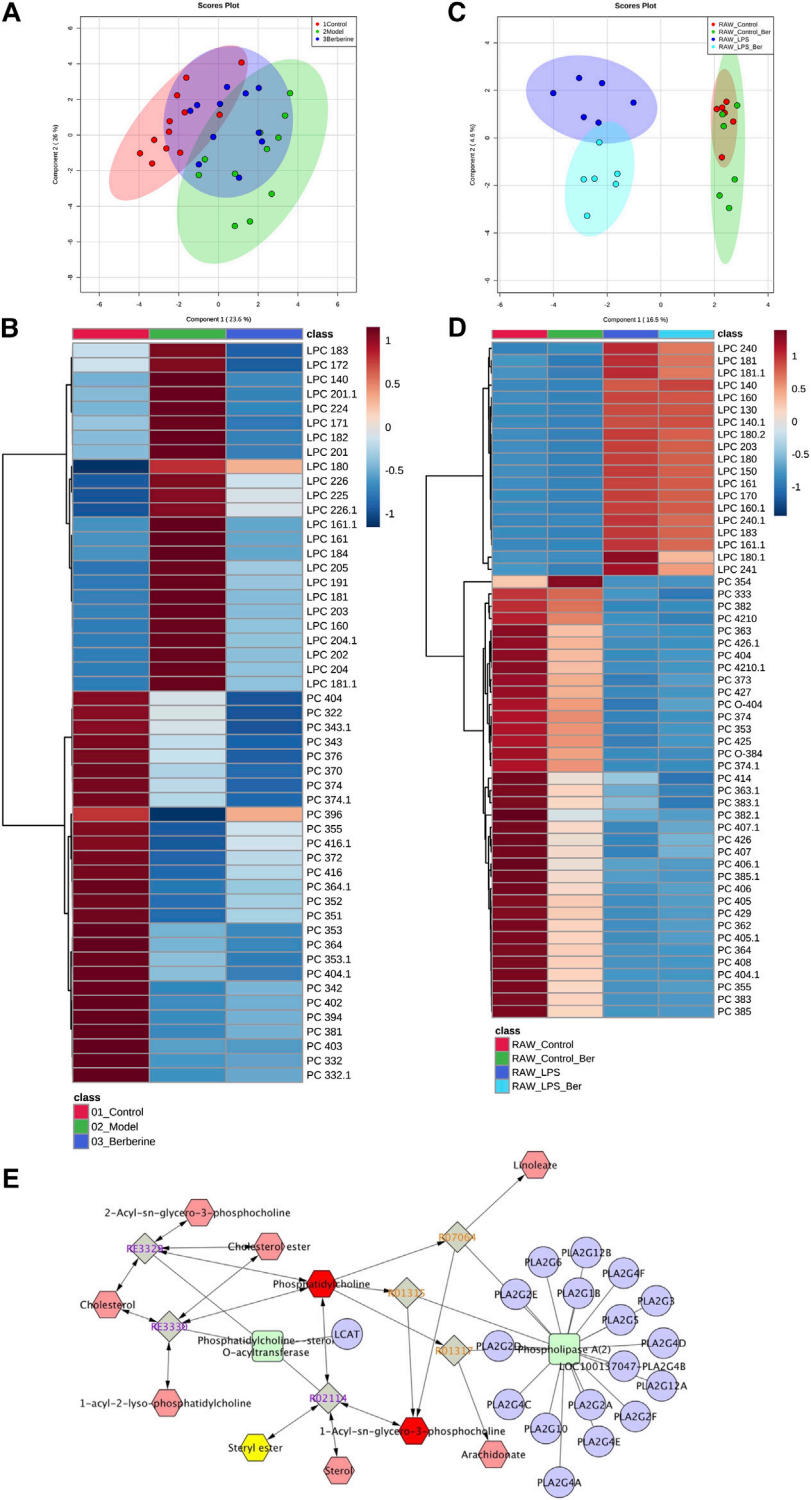
Berberine reduces the LPC level in DSS-treated colitis and LPS-stimulated RAW 264.7 cells. **(A)** PCA score plot of berberine treatment based on lipidomics profiling of DSS-induced colitis mice serum (merged using negative and positive ionization modes, ESI −/ESI +). **(B)** Heat map of PC and LPC profiles in DSS-induced colitis mice serum. **(C)** PCA score plot of berberine treatment based on lipidomics profiling of LPS-treated RAW cells. **(D)** Heat map of PC and LPC profiles in LPS-treated RAW cells. **(E)** KEGG pathway map of the PC and LPC metabolism.

We first investigated the effect of berberine on lipid metabolism in experimental colitic mice. DSS treatment caused a decrease in body weight, colon shortening, and increase in stool consistency as well as colonic bleeding. In contrast, treatment of berberine significantly ameliorated colon tissue injury and inflammatory responses in DSS-induced colitis mice (Supplementary Figure S1). To characterize and compare the lipid profiles of control, model, and berberine groups, biological samples including serum and colon tissues were collected and analyzed. Initially, we performed a lipidomics analysis of mouse serum to determine the changes in systemic lipid profiles. The score plot exhibited well-separated trends among the control group, model group, and berberine treatment group (Figure 1A). The heat map plot revealed that some LPC species significantly increased and PC species reduced in the model group compared with the control group (Figure 1B). Moreover, we found berberine treatment significantly reduced LPC and increased PC. This result indicated that berberine regulated the serum LPC level and PC level in DSS-induced colitis in mice.

Next, we studied the effects of berberine on inflammatory responses in LPS-treated RAW 264.7 macrophages (Qian et al., 2015). To investigate the regulatory effects of berberine on LPC species, we also determined the impact of berberine treatment on LPC profiles in LPS-treated RAW 264.7 macrophages. We first determined the anti-inflammatory effects of berberine on RAW 264.7 under pro-inflammatory stimulations (Supplementary Figures S2, S3). Berberine suppressed the expression of pro-inflammatory genes expression including *tnf-alpha*, *nos2*, *il6*, and *cxcl10* in the LPS-stimulated RAW 264.7 macrophages. Based on the results of cytotoxicity and anti-inflammatory effects of berberine on RAW 264.7 cells, berberine treatment at a concentration of 10 μM was chosen for lipidomics study. In RAW 264.7 cells, the score plot displayed clear classification among the control group, model group, and berberine treatment group, indicating that pro-inflammatory stimulations by LPS affect total lipid profiles. Berberine effectively reduced the elevated LPC levels in LPS-treated RAW 264.7 cells (Figures 1C,D). Subsequently, Cytoscape encoded with the KEGG database was used to address the related metabolic pathways of LPC. We found that phospholipase A2α is mainly responsible for the production of LPC (Figure 1E). Consistently, previous experimental findings have shown the involvement of phospholipase A2α in regulating inflammatory responses (Sun et al., 2010). Therefore, berberine treatment may inhibit phospholipase A2α activity in both DSS-treated colitis and LPS-stimulated RAW 264.7 cells.

### Berberine Regulates PLA2G4A Dysfunction in Dextran Sulfate Sodium–Treated Colitis and Lipopolysaccharides-Stimulated RAW 264.7 Cells

**FIGURE 2:**
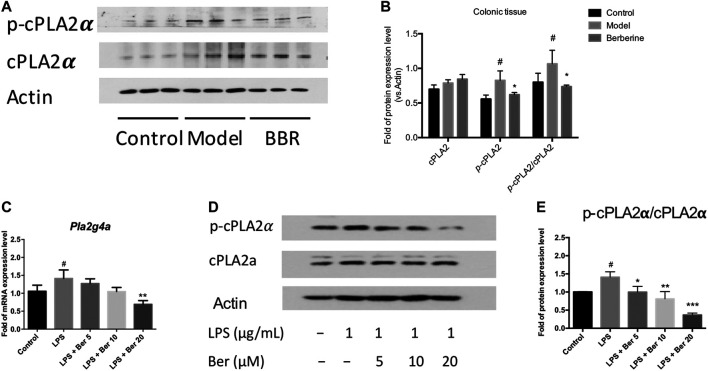
Berberine regulates PLA2G4A (cPLA2α) dysfunction in DSS-treated colitis and LPS-stimulated RAW 264.7 cells. **(A,B)** Immunoblot and the semi-quantitative results of cPLA2α and p-cPLA2α in colon tissues of berberine treatment on DSS-induced colitis mice. **(C)** *Pla2g4a* mRNA expression level of berberine treatment on LPS-treated RAW cells. **(D,E)** Immunoblot and the semi-quantitative results of cPLA2α and p-cPLA2α of berberine treatment on LPS-treated RAW cells. Data are given in means ± SD. *^, #^ *p* < 0.05, ** ^,##^ *p* < 0.01, and ***^, ###^ *p* < 0.001. # indicates comparisons between the control group and model group or LPS group. * indicates comparisons between the berberine group and model group or LPS group.

The cytosolic phospholipase A2α (PLA2G4A) activity has been shown to be increased in the colon biopsies of UC patients (Murase et al., 2016; Murase et al., 2017). Herein, we examined the expression of PLA2G4A and the effect of berberine on the PLA2G4A in experimental colitic mice. The phosphorylated forms of PLA2G4A (p-cPLA2α) was found up-regulated in the colon tissues of DSS-induced colitis mice and suppressed by berberine treatment (Figures 2A,B). Next, we examined the mRNA and protein expression levels of PLA2G4A in the RAW 264.7 macrophages under inflammatory conditions. The mRNA expression level of *Pla2g4a* and the protein expression level of *p*-cPLA2α all significantly increased after LPS stimulation. Berberine suppressed the up-regulation of PLA2G4A in LPS-stimulated RAW 264.7 macrophages in a dose-dependent manner (Figures 2C–E). Since PLA2G4A (cPLA2α) has been shown to be associated with the inflammation and PLA2G4A inhibitors significantly suppressed inflammatory responses in collagen-induced arthritis (Feuerherm et al., 2019), we wondered whether berberine suppresses the inflammatory responses by regulating PLA2G4A dysfunction.

### Berberine Suppresses Inflammation via Regulating PLA2G4A Activity in Lipopolysaccharides-Stimulated RAW 264.7 Cells

**FIGURE 3:**
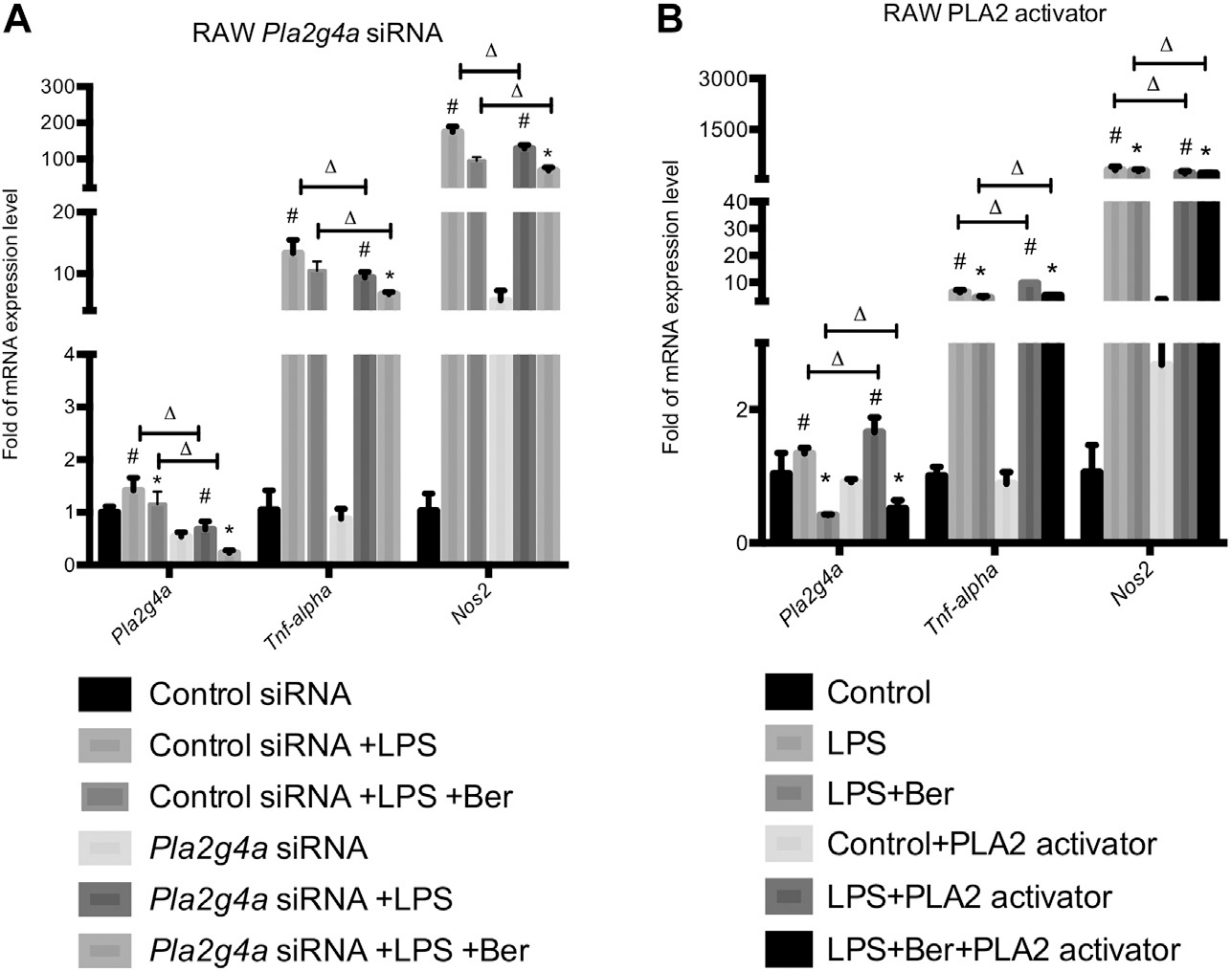
Berberine suppresses inflammation via regulating PLA2G4A activity in RAW 264.7 cells. **(A)** The mRNA expression level of Pla2g4a, Tnf-alpha, and Nos2 in RAW cells transfected with Pla2g4a siRNA. **(B)** The mRNA expression level of Pla2g4a, Tnf-alpha, and Nos2 in RAW cells treated with PLA2 activator. *comparisons between the LPS group and treatment group; # comparison between the LPS group and control group; ∆ comparisons in the normal group and knockdown/overexpressed models. Data are represented in means + SD (*n* = 4–6 independent measurements). *^, #, ∆^ *p* < 0.05, **^, ##, ∆∆^ *p* < 0.01, and ***^, ###, ∆∆∆^ *p* < 0.001.

Neon^®^ Transfection System was used to establish transfection models using *Pla2g4a* and control siRNA. Together with the transfection models, a phospholipase A2 (PLA2) activator, namely, sc-3034 (from PLA2 activating protein PLAP, 50 μg/ml) was used for activating PLA2G4A. Subsequently, we determined the expression levels of pro-inflammatory genes in knockdown and activating models of PLA2G4A RAW 264.7. We found the expression of pro-inflammatory gene levels decreased in the *Pla2g4a* siRNA model but increased by sc-3034 treatment. Meanwhile, we found that knockdown of *Pla2g4a* promoted berberine-induced suppression of pro-inflammatory genes in LPS-activated RAW 264.7 cells. In contrast, activation of PLA2G4A by sc-3034 suppressed the anti-inflammatory effects of berberine on LPS-stimulated RAW 264.7 cells (Figures 3A,B). Therefore, the anti-inflammatory properties of berberine are PLA2G4A-dependent in RAW 264.7 cells.

### Berberine Directly Inhibits PLA2G4A Activity

**FIGURE 4:**
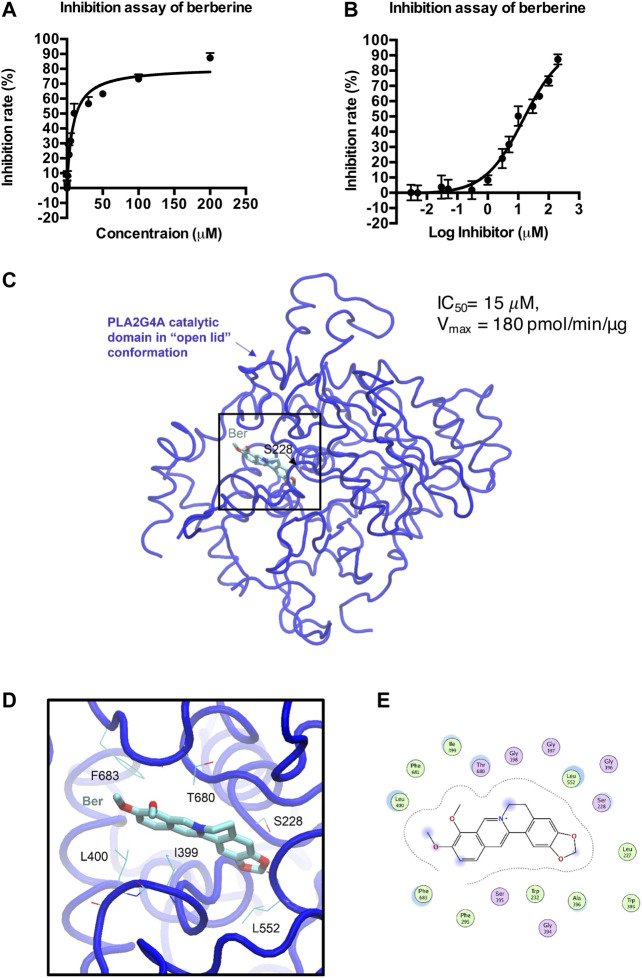
Docking model of berberine (Ber) and PLA2G4A catalytic domain. **(A,B)** The inhibition rate (%) of berberine on PLA2G4A activity. **(C)** The overall 3D structure of the berberine–PLA2G4A complex. The backbone of protein is rendered in tube and colored in blue. Berberine is rendered in stick and colored by element. The nucleophilic residue serine (S228)is rendered inline and colored by element. **(D)** A close view of the active site binding with berberine. Key residues interacted with berberine are rendered inline and colored by element. **(E)** The 2D protein–ligand interaction diagram of berberine–PLA2G4A complex. Protein residues are rendered in circles and colored based on their properties: green, hydrophobic residue; purple, polar residue.

We then determined whether berberine directly binds to PLA2G4A by using a human PLA2G4A protein assay. Berberine exhibited an IC_50_ = 15 μM on PLA2G4A activity (Figures 4A,B). The crystal structure of both the C2- and the catalytic domain of PLA2G4A was published in 1999 (PDB ID: 1CJY) (Dessen et al., 1999). However, in this structure, the catalytic domain is ligand-free, and the active site was covered by the lid (residue 396–461) (Supplementary Figure S4), which makes it unsuitable for docking study. Meanwhile, the catalytic domain with the “open lid” conformation of another member in the phospholipase A2 group IV, PLA2G4D, was resolved in 2016 (PDB ID: 5IXC) (Wang et al., 2016a). The lid in PLA2G4D (residue 530–575) was rotated, and the active site was occupied by a substrate-like inhibitor, methyl gamma-linolenyl fluorophosphonate (Supplementary Figure S4). Therefore, to understand the binding mode of berberine on PLA2G4A, we performed molecular docking based on previous structural studies of PLA2G1D. By aligning the sequences and main-chain atom positions in these two structures (Supplementary Figure S5), we built a putative model of the “open lid” conformation of PLA2G4A with homology modeling (Supplementary Figure S6A). The model quality was reasonably good, and the backbone dihedral angles of most residues were within favorable and allowed regions (Supplementary Figure S6B). Based on this putative, “open lid,” and holo-structure of PLA2G4A, we performed the docking study of berberine. The docking results showed berberine could insert into the active site of the catalytic domain of PLA2G4A (Figures 4C–E). The best docking pose was with a binding energy of −7.02 kcal/mol suggesting that berberine may have favorable interactions with the PLA2G4A catalytic domain. Interaction analysis based on structural observation indicated that residues I399, L400, L552, T680, and F683 may have hydrophobic interactions with berberine. However, neither hydrogen bond nor salt bridge interactions could be observed from the docking complex. Taken together, the docking study showed that berberine could be a moderate binder of the PLA2G4A catalytic domain.

### Berberine Inhibits PLA2G4A Activity via Mitogen-Activated Protein Kinase/c-Jun N-Terminal Kinase Signaling Pathway

**FIGURE 5:**
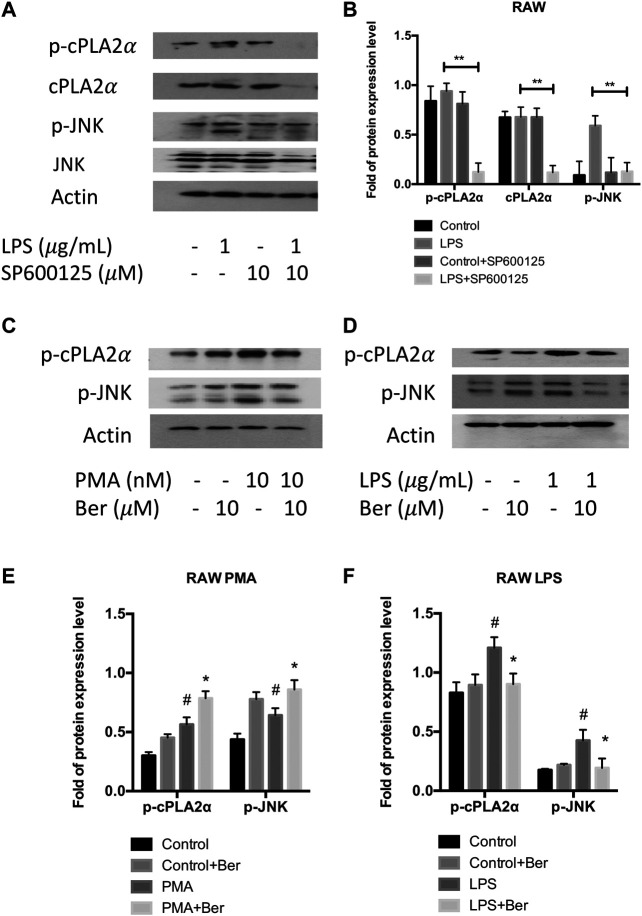
Berberine inhibits PLA2G4A activity via the MAPK/JNK signaling pathway. **(A,B)** The immunoblot and semi-quantification results of cPLA2α, p-cPLA2α, JNK, and p-JNK in RAW cells treated with JNK inhibitor SP600125. * indicates comparisons in the model group in the absence or presence of SP600125. **(C,E)** The immunoblot and semi-quantification results of *p*-cPLA2α and *p*-JNK in RAW cells treated with berberine and JNK activator PMA. **(D,F)** The immunoblot and semi-quantification results of p-cPLA2α and p-JNK in inflamed RAW cells treated with berberine. # indicates comparisons between control group and PMA or LPS group. * indicates comparisons of PMA/LPS treatment in the absence or presence of berberine treatment. ^*, #^ *p* < 0.05, ^**, ##^ *p* < 0.01, and ^***, ###^ *p* < 0.001.

**FIGURE 6:**
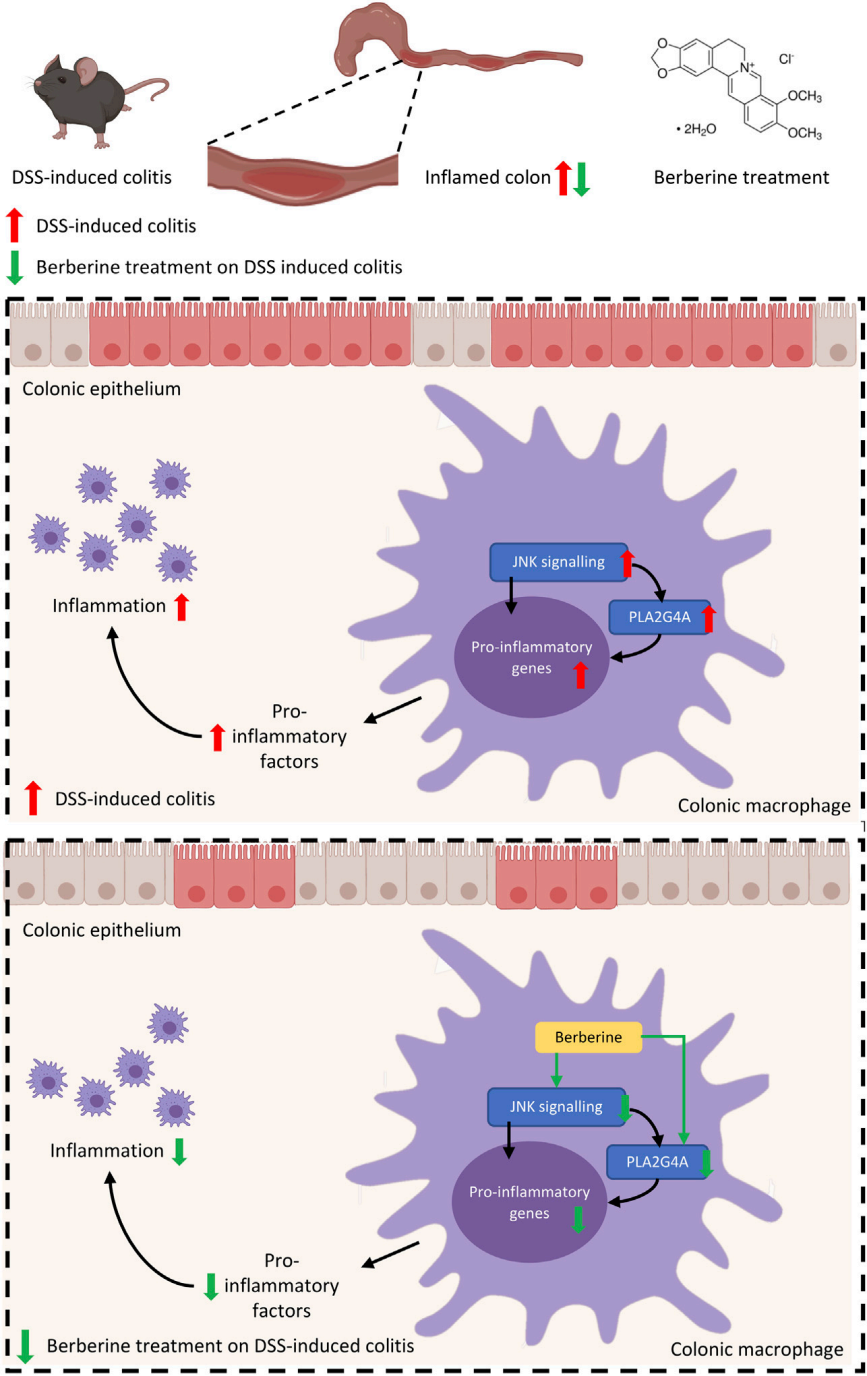
Mechanisms of berberine anti-inflammatory effects in experimental colitis are mediated by PLA2G4A in colonic macrophages.

Since previous studies have shown PLA2G4A is regulated via the MAPK/JNK signaling pathway (Casas et al., 2009), we used JNK inhibitor SP600125 to investigate whether PLA2G4A is mediated by the JNK signaling pathway in RAW 264.7 cells. The protein expression levels of *p*-JNK and *p*-cPLAα were significantly increased in LPS-stimulated RAW 264.7 cells, which was suppressed by SP600125 (10 μM) (Figures 5A,B). Next, we used a nonspecific JNK activator PMA to investigate whether berberine can regulate the dysfunction of PLA2G4A via the JNK signaling pathway. We revealed that *p*-JNK and *p*-cPLAα were up-regulated significantly after treating RAW with PMA (Figures 5C,E), whilst berberine treatment suppressed the up-regulation of p-JNK and p-cPLA2α in LPS-treated RAW 264.7 cells (Figures 5D,F). Combining the results together, berberine treatment regulates PLA2G4A dysfunction via both direct binding and regulation of the JNK signaling pathway in LPS-stimulated RAW 264.7 cells (Figure 6).

## Discussion and Conclusion

In this study, we have shown a novel phospholipid-related mechanism underlying the anti-inflammatory effects of berberine. First, we demonstrated the aberrant activation of PLA2G4A in inflammatory responses in both *in vivo* experimental murine colitis and *in vitro* RAW 264.7 macrophages. In these *in vivo* and *in vitro* models, we showed berberine treatment significantly reduces the phosphorylation of PLA2G4A. Second, we showed berberine suppressed the expression of pro-inflammatory genes by inhibiting PLA2G4A in these models. Mechanically, we revealed that berberine directly inhibits PLA2G4A activity and regulates PLA2G4A activation via the MAPK/JNK signaling pathway in inflammatory responses. Collectively, this study suggested berberine suppresses inflammatory responses through inhibition of PLA2G4A activity to ameliorate DSS-induced experimental colitis.

Numerous studies have revealed the anti-inflammatory properties of berberine in the digestive system (Habtemariam, 2016). The anti-inflammatory activity of berberine is mainly dependent on the inhibition of expression of pro-inflammatory factors including TNF-alpha, IL-6, IL-8, IFN-γ, and IL-1β (Zou et al., 2017). It has been reported the inhibitory effects of berberine on pro-inflammatory responses including TNF-alpha, IL-6, iNOS, and COX-2 were mediated through activation of AMP-activated protein kinase (AMPK) (Jeong et al., 2009). Moreover, studies have shown berberine exerts anti-inflammatory effects on DSS-induced colitis via Akt, NF-κB, and MAPK dependent signaling pathways (Amasheh et al., 2010; Remppis et al., 2010). In this study, we further investigated the pharmacological mechanism of berberine treatment in the experimental colitis. Lysophospholipids have attracted increased attention due to their importance in maintaining the function of the intestinal mucus barrier (Wang et al., 2018). First, PC and LPC contents are intensely associated with UC in both human and animal colitis models. Patients with UC have significantly increased serum LPC content and an increased LPC/PC ratio in the colonic mucus in both remission and active disease states (Ehehalt et al., 2004; Franzosa et al., 2019). In animal models of UC, LPC (16:0) has been found elevated in the serum of TNBS-induced colitis mice (Zhang et al., 2012) and DSS-induced colitis mice (Wang et al., 2016b) through untargeted metabolomics profiling. Second, LPC may contribute to the pathophysiological progression of UC. PC has been shown with anti-inflammatory effects on the intestinal mucosal barrier (Schneider et al., 2010), while LPC has been revealed with pro-inflammatory phenotype on macrophages (Qin et al., 2014). The increased LPC/PC ratio is due to the elevated activity of catalyzed enzyme phospholipase A2α. Recent studies have indicated that cytosolic phospholipase A2α up-regulation is associated with the severity of inflammation (Gil-de-Gomez et al., 2014; Ashley et al., 2016) and the development of murine colitis (Rosengarten et al., 2016). Herein, phospholipase A2 is considered as a potential therapeutic target for UC. Our results confirmed that berberine, as a potent anti-inflammatory agent on UC, regulates the LPC content and the PLA2G4A expression in experimental colitis mice. Similarly, one study also showed berberine (50 mg/kg) protects against LPS-induced lung injury and inhibits cytosolic phospholipase A2α in lung tissue, whereas this study lacks mechanistic insights into the berberine action on PLA2G4A (Zhang et al., 2008). In contrast, our study demonstrated the mechanisms of therapeutic effects of berberine on murine colitis is mediated by inhibiting cytosolic phospholipase A2α via direct binding and the MAPK/JNK signaling pathway.

Macrophages are responsible for innate immune responses and play an important role in mediating colonic mucosal inflammation in IBD patients (Steinbach and Plevy, 2013). Defined by the broad terms of pro-inflammatory (M1) and anti-inflammatory (M2) phenotypes, macrophages are activated toward different phenotypes depending on the stimuli (Murray et al., 2014). The colonic macrophages are mainly polarized to M1 pro-inflammatory phenotype in the colon and linked to disease severity in IBD (Lissner et al., 2015). The anti-inflammatory effects of berberine on colitis are mediated by multiple signaling pathways, such as AKT, AMPK, and MAPK/JNK signaling pathways (Cui et al., 2009; Jeong et al., 2009; Amasheh et al., 2010; Zhou et al., 2015). Nevertheless, the mechanisms of berberine treatment in suppressing inflammation in macrophages in IBD are not fully understood. On the one hand, considering berberine directly binds to secretory phospholipase A2 (Chandra et al., 2011), berberine may directly inhibit cytosolic phospholipase A2 activity. Using human PLA2G4A protein assay, we found that berberine directly inhibits its activity. On the other hand, by addressing the related references (Casas et al., 2009; Guijas et al., 2012; Lee et al., 2013), we found cytosolic phospholipase A2α is also possibly regulated by the JNK signaling pathway in IBD. Additionally, JNK is found to be up-regulated in colon tissues of DSS-induced colitis mice (Hollenbach et al., 2004; Gao et al., 2016) and inflamed mucosa of IBD patients (Docena et al., 2010; Zhou et al., 2017). JNK inhibitor has also been shown for protective effects on experimental colitis in mice (Docena et al., 2010; Reinecke et al., 2012). Besides the direct binding, we have shown that the inhibitory effect of berberine on cytosolic phospholipase A2α activity is also dependent on the MAPK/JNK signaling pathway in RAW 264.7 macrophages.

There are several limitations to this study. First, to examine the role of PLA2G4A in mediating the inflammatory responses and whether the anti-inflammatory properties of berberine treatment are through inhibition of PLA2G4A, we have used *Pla2g4a* siRNA to knock down the expression of cytosolic phospholipase A2α in *in vitro* cell models but not in *in vivo* animal models. Second, due to the lack of specific JNK activator, we used a general MAPK activator PMA to induce the activation of MAPK/JNK signaling to determine whether the inhibitory effects of berberine treatment on cytosolic phospholipase A2α are mediated by MAPK/JNK. In the future, we will establish colon-specific *Pla2g4a* knockout mice to further understand the role of colonic *Pla2g4a* in the inflammatory responses of IBD. Moreover, we will perform a screening assay of PLA2G4A to investigate the inhibitory activity of bioactive components from herbal extracts on PLA2G4A in order to provide potential therapeutic strategies for IBD.

In conclusion, this study demonstrated that berberine suppresses colonic inflammatory responses through inhibition of PLA2G4A activity to ameliorate DSS-induced experimental colitis, which enhances our understanding on the pharmacological mechanism of berberine treatment on ulcerative colitis from a perspective of lipid metabolism and signaling. This study highlights the therapeutic potential of targeting cytosolic phospholipase A2a dysfunction in the management of IBD.

## Data Availability Statement

The raw data supporting the conclusions of this article will be made available by the authors, without undue reservation, to any qualified researcher.

## Ethics Statement

The animal study was reviewed and approved by Animal Care Ethics Committee, Hong Kong Baptist University.

## Author Contributions

Z-XB, H-TX, and C-YL designed the study and revised the manuscript. LXZ, C-YL, and P-GW conducted the animal study, cell study and wrote the manuscript. TH conducted molecular docking study. X-JH, Z-WN, and LXZ performed the lipidomics analysis. LZ, SF, HK, and HW provided technical support and advice toward this study.

## Funding

This work was kindly funded by National Natural Science Foundation of China (Grants No. 81973538 and 81560676), Key-Area Research and Development Program of Guangdong Province (Grants No. 2020B1111110003), Shenzhen Science and Technology Innovation Committee Grant (Grants No. JCYJ20170413170320959 and JCYJ20190808164201654), and Logan Charitable Foundation.

## Conflict of Interest

The authors declare that the research was conducted in the absence of any commercial or financial relationships that could be construed as a potential conflict of interest.

## Abbreviations

BBR, berberine; DSS, dextran sulfate sodium; LPC, lysophosphatidylcholine; PC, phosphatidylcholine; PLA2, phospholipase A2; UC, ulcerative colitis.
