# Supplementary material for: Berberine Suppresses Colonic Inflammation in Dextran Sulfate Sodium–Induced Murine Colitis Through Inhibition of Cytosolic Phospholipase A2 Activity
Source: Front Pharmacol. 2020 Nov 19;11:576496. doi: 10.3389/fphar.2020.576496 (PMC7919193; doi:10.3389/fphar.2020.576496)
Supplement: Supplementary file 1 [file DataSheet1_v1.docx]

**Berberine suppresses colonic inflammation in DSS-induced murine colitis through inhibition of cytosolic phospholipase A2 activity**

**Supplement Figure**


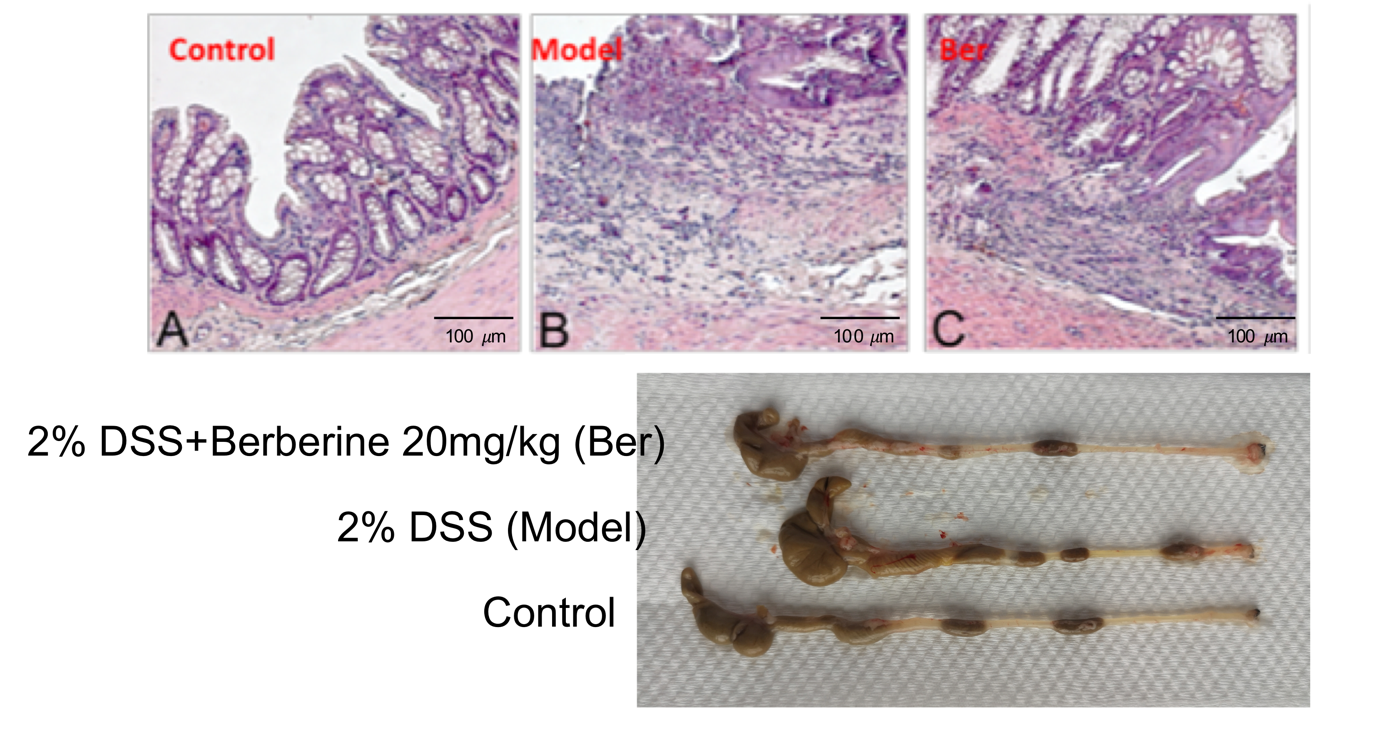


Supplement Figure S1 H&E histology plot and the length of the colon tissues of DSS-induced colitis and berberine treatment in C57BL6 mice.


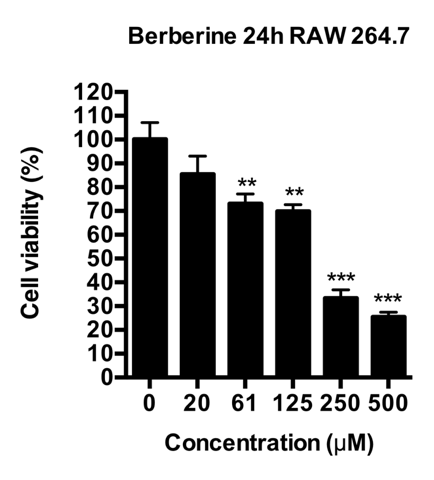


Supplement Figure S2 The cytotoxicity result of berberine on RAW 264.7 cells for 24h using the MTT method. Berberine at a concentration of 20 μM exhibited no significant difference in cell viability. * p < 0.05, ** p < 0.01 and *** p < 0.001. * indicates comparisons between control group and berberine treatment group.


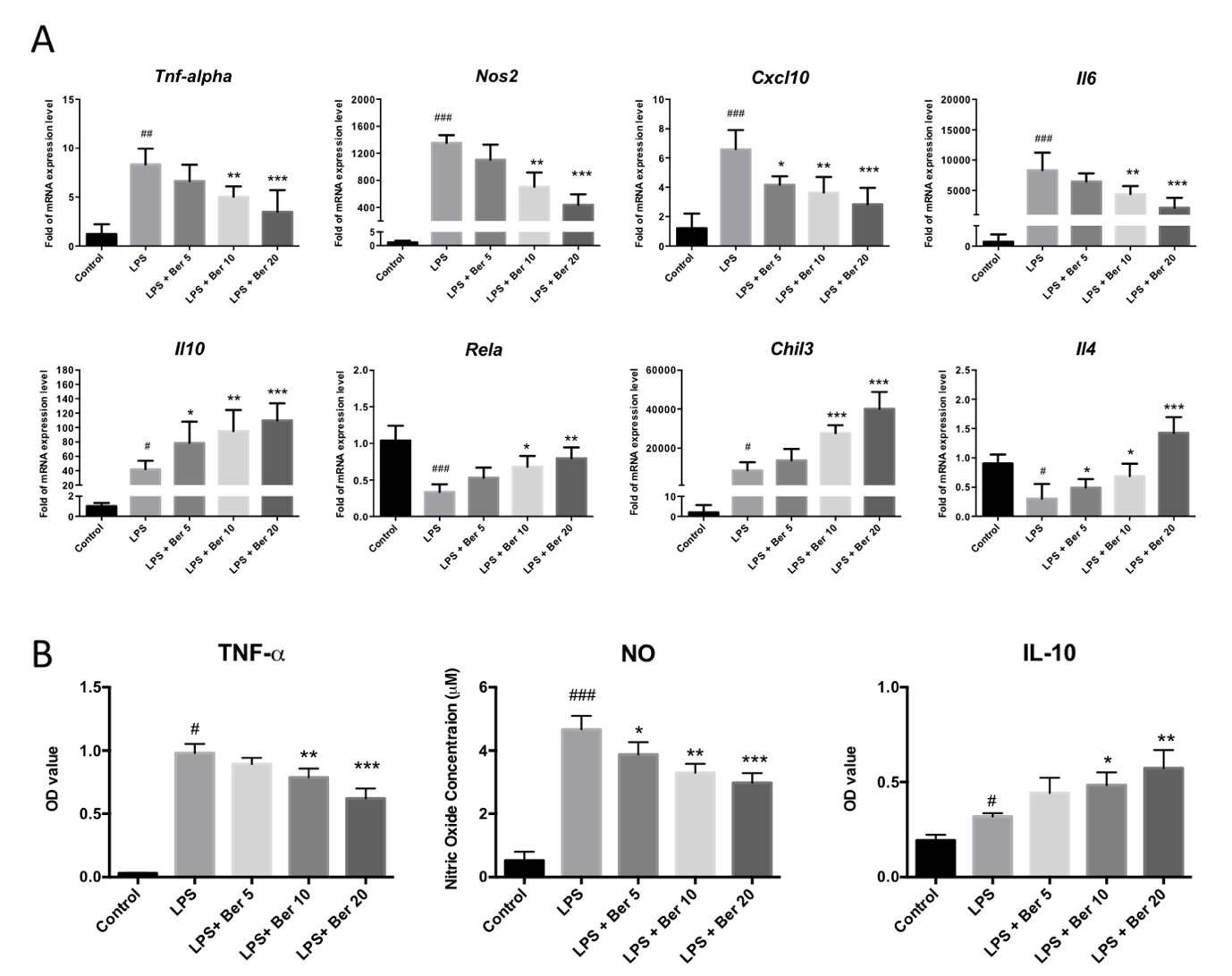


Supplement Figure S3 RAW 264.7 macrophage was treated in the absence or presence of LPS (1 μg/mL), berberine (5, 10, 20 μM). (A) Effect of the berberine treatment on mRNA expression of selected M1 and M2 markers. The M1 markers include *Tnf-alpha, Nos2, Il6* and *Cxcl10*, and M2 markers include *Il10*, *Rela*, *Chil3* and *Il4*. (B) Effect of the berberine treatment on TNF-α, nitric oxide, IL-10 measured in the culture medium. Data are the means + S.D. (n=4-6 independent measurements). p < 0.05, ** p < 0.01 and *** p < 0.001;


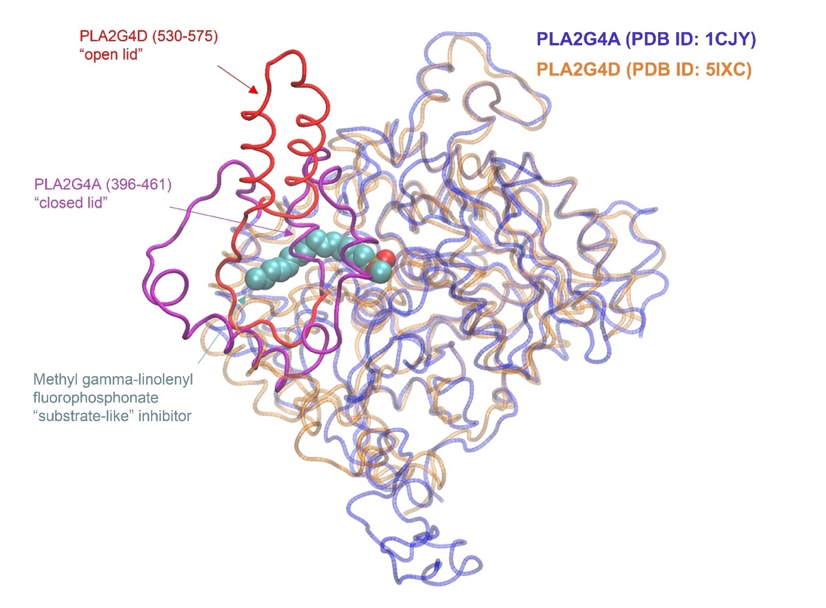


Supplement Figure S4 The comparison of “open lid” and “closed lid” catalytic domain structures in two phospholipase A2 enzymes. The “closed lid” catalytic domain structure was modeled based on the crystal structure of phospholipase A2 group IVA (PLA2G4A) (PDB ID: 1CJY) and colored in blue. The “open lid” catalytic domain structure was modeled based on the crystal structure of phospholipase A2 group IVD (PLA2G4D) (PDB ID: 5IXC) and colored in orange. The “closed lid” was highlighted with purple color in PLA2G4A, residue 396-461; while the “open lid” was highlighted with red color in PLA2G4D, residue 530-575. The methyl gamma-linolenyl fluorophosphonate, a substrate-like inhibitor of PLA2G4D, which was rendered in van der Waals sphere and colored by element, was shown to demonstrate the active site of the catalytic domain.


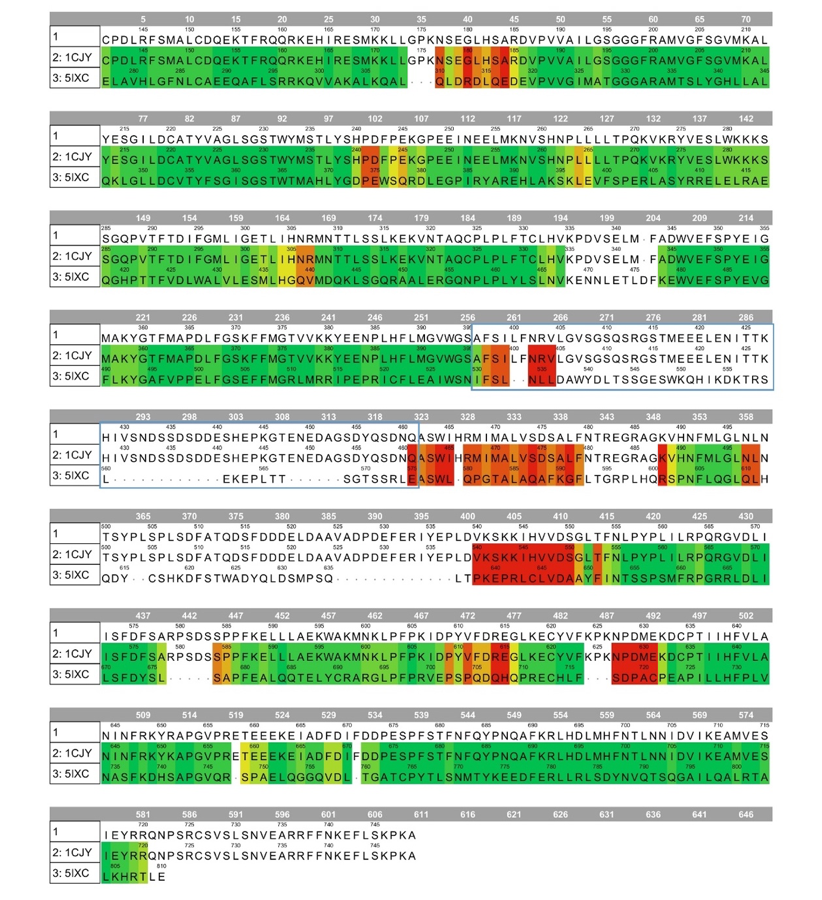


Supplementary Figure S5 The alignment between PLA2G4A and PLA2G4D catalytic domain structure. The catalytic domains of PLA2G4A (residue 141 to 749) and PLA2G4D (residue 277 to 810) were aligned based on the structure superposition (1CJY, PLA2G4A; 5IXC, PLA2G4D). All the residues were colored based on main-chain atom RMSD: the green color means low RMSD, while the red color represents high RMSD. The “closed” and “open” lids were highlighted in the blue rectangle.


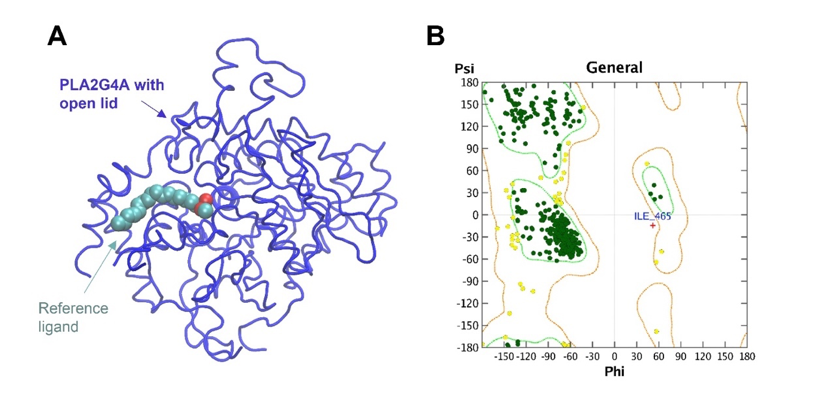


Supplementary Figure S6. The putative model of the “open lid” conformation of PLA2G4A catalytic domain. (A) The overall 3D structure of “open lid” conformation of PLA2G4A catalytic domain. The backbone of protein was rendered in tube and colored in blue. The methyl gamma-linolenyl fluorophosphonate, a substrate-like inhibitor of PLA2G4D, which was rendered in van der Waals sphere and colored by the element, was used as reference ligand to indicate the active side. (B) The phi-psi plot of the putative model of PLA2G4A “open lid” conformation. Each dot is corresponding to one residue. The green circle dot means that the dihedral angles of that residue are in favorable region. The yellow circle dot means that the dihedral angles of that residue are in the allowed region. The red cross dot means that the dihedral angle is an outlier.

Supplement Table S1 Primers lists for targets analysed by RT-qPCR (F, forward; R, reverse)

| Name | Sequence (5’ to 3’). | Tm (salt) | Product size |
| --- | --- | --- | --- |
| Nos2.mf | CAGGTCTTTGACGCTCGGAA | 60 | 167 |
| Nos2.mr | GCCTGAAGTCATGTTTGCCG | 60 | 167 |
| Il-6.mf | GTTCTCTGGGAAATCGTGGA | 60 | 160 |
| Il-6.mr | TGTACTCCAGGTAGCTA | 60 | 160 |
| Tnfa.mf | GGTGCCTATGTCTCAGCCTC | 60 | 177 |
| Tnfa.mr | GCTCCTCCACTTGGTGGTTT | 60 | 177 |
| Cxcl10.mf | ATGACGGGCCAGTGAGAATG | 60 | 249 |
| Cxcl10.mr | GAGGCTCTCTGCTGTCCATC | 60 | 249 |
| Il-10.mf | ATCGATTTCTCCCCTGTGAA | 60 | 328 |
| Il-10.mr | CACACTGCAGGTGTTTTAGCTT | 60 | 328 |
| Rela.mf | GAACCTGGGGATCCAGTGTG | 60 | 266 |
| Rela.mr | AGTTCCGGTTTACTCGGCAG | 60 | 266 |
| Chil3.mf | AAGCTCTCCAGAAGCAATCCT | 59 | 187 |
| Chil3.mr | GAGTACACAGGCAGGGGTCA | 61 | 187 |
| Il4.mf | CCATATCCACGGATGCGACA | 60 | 131 |
| Il4.mr | CTGTGGTGTTCTTCGTTGCTG | 60 | 131 |
| Pla2g4a.mf | ACGTGCCACCAAAGTAACCA | 60 | 97 |
| Pla2g4a.mr | CCTGCTGTCAGGGGTTGTAG | 60 | 97 |
| Actin.mf | CTGTCCCTGTATGCCTCTG | 60 | 218 |
| Actin.mr | ATGTCACGCACGATTTCC | 60 | 218 |
